# Close to the Comfort Zone: Stakeholders’ Perspectives on Implementing Leisure Activities in Dementia and Eldercare

**DOI:** 10.3390/bs15030347

**Published:** 2025-03-12

**Authors:** Golnaz Atefi, Hannah L. Christie, Marjolein E. de Vugt, Michael P. Craven

**Affiliations:** 1 Department of Psychiatry and Neuropsychology, Alzheimer Centre Limburg, Maastricht University, 6229 ET Maastricht, The Netherlands; 2 School of Population Health, Royal College of Surgeons in Ireland, D02 YN77 Dublin, Ireland; 3 NIHR MindTech HealthTech Research Centre, Institute of Mental Health, University of Nottingham, Nottingham NG7 2RD, UK; 4 Human Factors Research Group, Faculty of Engineering, University of Nottingham, Nottingham NG7 2RD, UK

**Keywords:** eldercare, dementia care, social isolation, leisure activity, implementation readiness, inclusive design, social well-being

## Abstract

Background: This study aimed to explore stakeholders’ perspectives on implementing non-digital leisure activities to promote social interaction in dementia and eldercare settings. Methods: A secondary analysis of online semi-structured interviews was conducted, focusing on nine stakeholders with expertise in dementia and eldercare. The data collected were analyzed using inductive qualitative thematic analysis to identify key themes and insights. Results: Three key themes emerged, emphasizing inclusivity, usability, and context. Stakeholders highlighted the importance of tailored activities, ease of use, and privacy. Challenges included resource allocation and availability. The findings underscore the significance of supporting innovations in both digital and non-digital leisure activities within dementia and eldercare settings. Clinicians and policymakers should consider integrating inclusive activities into care plans to enhance social interaction for older adults. Future research should focus on identifying optimal levels of engagement and evaluating the effectiveness of leisure activities in promoting well-being among older adults in diverse settings. Conclusion: Despite current limitations, stakeholders affirmed the value of non-digital leisure activities, such as board games, for enhancing social interaction and well-being in dementia and eldercare settings. Integrating non-digital and digital activities was seen as promising for meeting diverse needs.

## 1. Introduction

### 1.1. Aging and Social Isolation

With global demographics trending towards an aging population, it is becoming increasingly essential to prioritize the improvement of well-being and quality of life for older adults (Owen et al., 2021; Clarke et al., 2020). As aging progresses, various challenges emerge, with social functioning often affected by increased risks of social isolation and decreased opportunities for meaningful social interaction (Y. R. Chen & Schulz, 2016). Social well-being holds particular significance in older age, as feelings of loneliness and disconnection can lead to various health risks (Jaremka et al., 2013; Steptoe et al., 2013). These include an increased risk of developing symptoms of dementia and mild cognitive impairment in older adults (Sutin et al., 2018), as well as depressive symptoms in people with dementia (Tsai et al., 2020). Further, challenges of social well-being are particularly exacerbated when transitioning to nursing homes as relocating to a nursing home involves accepting the loss of familiar surroundings and adapting to a new environment (Altıntaş et al., 2017).

#### 1.1.1. Leisure Activity

Numerous approaches have proven to be effective in addressing social functioning, with leisure activities standing out as particularly noteworthy in addressing social isolation in older adults with and without dementia (Fakoya et al., 2020; Verghese et al., 2003). Leisure activities, defined as voluntary engagements during free time, aim to provide participants with entertainment, relaxation, vitality, and stimulation (Pressman et al., 2009). Engaging in leisure activities has been identified as a factor in promoting well-being (Takiguchi et al., 2022). Specifically, in the context of aging, leisure activities have been reported to have a positive association with cognitive function, physical function, and mental health among older adults and the elderly population (Sala et al., 2019). In the context of dementia, engagement in leisure activities can promote a sense of belonging and identity (Russell et al., 2022). Further, adaptation to the nursing home can be facilitated by leisure activities and their role in promoting motivation and enhancing relatedness (Altıntaş et al., 2017).

Leisure activities, depending on the context, can serve multiple purposes, offering both practical and hedonic benefits (Li et al., 2021). While some activities primarily provide entertainment, others offer deeper engagement, fostering a sense of belonging, cognitive stimulation, and social interaction.

#### 1.1.2. Psychological and Hedonic Leisure Activities in Aging

Psychological and hedonic leisure activities are activities that engage cognitive abilities and offer emotional satisfaction (Niedderer et al., 2022). Examples include board games, creative arts, storytelling, reading, solving puzzles, or listening to music. These activities differ from physically oriented leisure activities, such as hiking or gardening, which focus on physical engagement, and from pragmatic activities, which aim to achieve practical benefits, such as cooking. By promoting mental engagement and enjoyment, psychological and hedonic leisure activities can enhance well-being and social connectedness (Niedderer et al., 2022). Maintaining an appropriate level of engagement and personal involvement in psychological and hedonic leisure activities is positively associated with greater benefits across various domains, such as health, emotional well-being, social interactions, and self-realization (Li et al., 2021). Therefore, considering the risk of social isolation in later life, this aspect of leisure activity holds significant importance, especially as they do not demand significant physical or psychological effort from individuals. With the ongoing digitalization of dementia and eldercare, digital leisure is becoming more prevalent. However, even within the context of digital leisure, social interaction serves as the primary predictor for adherence and longer engagement for older adults. It highlights the importance and necessity of social connectedness in this demographic (De Schutter, 2010). Previous research reviewed the role of technology, such as digital life storybooks, in contributing to enhancing social well-being in long-term care settings (Budak et al., 2021). Although digital innovations hold promise for supporting the social well-being of older adults, it is important to note that not everyone, especially those residing in nursing homes, may have the capability or willingness to access or use digital resources (Boots et al., 2016; Arighi et al., 2021). Therefore, recognizing the significance of social well-being among older adults and acknowledging the risk of digital exclusion, gaining further insights into the implementation of non-digital leisure activities could offer an alternative and inclusive approach to engaging vulnerable older adults in leisure and meaningful activities (Niedderer et al., 2022; Dartigues et al., 2013).

### 1.2. Present Study

Despite growing interest in digital interventions for dementia and eldercare, research on the practical implementation of non-digital leisure activities remains limited. This gap is concerning given the significant digital divide experienced by older adults, especially those with dementia, who may lack the resources or digital literacy to engage with these technologies (Arighi et al., 2021; Giebel, 2023). While prior studies have demonstrated their benefits (Noda et al., 2019; Giebel et al., 2024), there is a lack of knowledge regarding how these activities are integrated into care settings, the barriers they face, and their feasibility from the perspective of key stakeholders (Branco et al., 2017; Niedderer et al., 2022). Addressing this gap is essential to develop inclusive strategies that cater to the diverse needs of older adults in various care settings. Although person-centered care is well established in nursing homes, the feasibility and implementation of leisure activities remain largely influenced by stakeholders who develop, fund, and integrate these activities into care structures (Garratt et al., 2021). Policymakers, industry professionals, and clinicians can play a role in shaping the availability, accessibility, and sustainability of such initiatives (Hirt et al., 2021). Therefore, examining their perspectives provides critical insights into the systemic facilitators and barriers that affect the successful adoption of non-digital leisure activities in dementia and eldercare settings.

Addressing this knowledge gap, the present study seeks to answer the following research question: what are stakeholders’ views on implementing non-digital leisure activities to promote social interaction in dementia and eldercare settings? In this study, stakeholders were asked about the implementation of non-digital leisure activities that are not only enjoyable but also meaningful in promoting well-being and interpersonal connections.

## 2. Materials and Methods

The study received ethical approval from Maastricht University’s Medical Ethical Oversight Commission (approval number 2022-3176). We conducted a secondary analysis of qualitative interviews to further explore and deepen our understanding of the implementation of non-digital leisure activities. These interviews were originally conducted as part of a study that aimed to create a tool for assessing eHealth interventions for dementia by adapting an existing checklist, with insight from stakeholders. The study was based on the Nonadoption, Abandonment, and challenges to the Scale-Up, Spread, and Sustainability of Health and care technologies (NASSS) framework (Greenhalgh & Abimbola, 2019) which emphasizes the interplay between technological, social, organizational, and policy-related factors in the adoption and scaling of health and care technologies. The primary findings are reported elsewhere (Christie et al., 2024). Data were re-examined in the context of our research objectives to extract additional insights and perspectives. Thus, the present study aims to address the secondary research question: what are stakeholders’ views on implementing non-digital leisure activities to promote social interaction in dementia and eldercare settings?

The decision to focus on stakeholders rather than older adults themselves is due to the critical role stakeholders play in the development, implementation, and evaluation of leisure activities within care settings. Stakeholders, including clinicians, policymakers, and industry professionals, often act as intermediaries, shaping the opportunities and resources available to older adults. Understanding their perspectives is essential to addressing systemic and practical barriers to implementing meaningful activities. Although older adults’ direct experiences are indispensable (Ganann et al., 2022), this study serves as a necessary first step to identify structural and logistical challenges before future research incorporates their personal preferences and lived experiences.

### 2.1. Design

This study used secondary analysis to revisit qualitative data originally collected to explore stakeholder perspectives on implementing eHealth in dementia care. The study involved conducting online, semi-structured individual interviews guided by an interview questionnaire. Secondary analysis was chosen as it allowed for the extraction of additional insights from existing datasets, particularly when addressing the adjacent topic of implementing non-digital tools. For example, participants were asked about tools or activities that promote social interaction, which sometimes led to mentions of non-digital options. Although the original interview guide focused on implementing eHealth in dementia care, several questions indirectly prompted discussions on non-digital tools, and two questions specifically addressed the value of non-digital tools and leisure activities such as board games (Supplementary Materials). Relevant responses addressing non-digital leisure activities were categorized and analyzed separately, allowing for a distinction between these and discussions of digital tools. To ensure the reliability of the findings, transcripts were systematically reviewed to identify content explicitly or implicitly related to implementing non-digital tools and leisure activities. Additional details, along with the primary findings, are reported elsewhere (Christie et al., 2024), and the complete interview guide is available in Supplementary Materials. Further details of the analysis process are provided below.

### 2.2. Participants and Recruitment

Informed consent was obtained from all participants before their involvement in the study, ensuring that their privacy rights were carefully respected. Participants were recruited through targeted email invitations by authors A and B using professional networks and snowball sampling techniques. Eligible participants included stakeholders with expertise in dementia and eldercare who were actively involved in implementing care and support in clinical, policy, or research contexts. Recruitment aimed to ensure diverse perspectives across domains, including clinicians, policymakers, and researchers. Additionally, participants were recruited from different countries, including the United Kingdom, Canada, the Netherlands, Spain, and France, to capture diverse perspectives. A total of 15 individuals were contacted, with 13 responding positively, resulting in a final sample of 9 participants. Recruiting 8–10 participants is deemed appropriate based on previous recommendations for sample sizes in in-depth exploratory qualitative studies (Boddy, 2016). The reasons for non-participation included scheduling conflicts (n = 2) and misalignment with their current objectives (n = 2). Table 1 outlines the participants’ backgrounds and diverse areas of expertise present in this study.

Online individual semi-structured interviews were conducted between May and June 2022. As described in Christie et al. (2024), the recruitment process was designed to answer the original research question on eHealth implementation readiness and aligns with the scope of this secondary analysis.

### 2.3. Data Collection

Nine professionals participated in this interview study, meeting the inclusion criteria of being researchers, policymakers, clinicians, or other stakeholders in the fields of dementia and eldercare with experience in implementing care and support. Participants were included based on their professional expertise and their experience with the development, adoption, or implementation of (eHealth) interventions in dementia care, eldercare, or related fields. This approach was chosen to ensure that the study captured diverse perspectives on the systemic and organizational factors influencing the implementation of (non-)digital tools. The sole exclusion criteria were the unavailability of or lack of interest or non-response from potential participants. Participants had diverse backgrounds and expertise from the United Kingdom (n = 4), Canada (n = 1), Spain (n = 1), the Netherlands (n = 1), and France (n = 1). The interviews, conducted via MS Teams, were individually held by authors A and B, averaging 38 min in duration, and were audio-recorded and transcribed verbatim.

### 2.4. Data Analysis

The secondary analysis involved revisiting qualitative data originally collected for the parent study. Authors A and B conducted the interviews, including questions about implementing both digital and non-digital tools in dementia and eldercare settings (Supplementary Materials). Further details on the development of the interview guide are available elsewhere (Christie et al., 2024). This secondary analysis specifically targeted responses related to the implementation of non-digital tools such as board games that are meant to be enjoyable rather than therapeutic as subtypes of non-digital leisure activities. This approach allowed for exploring the perspective of stakeholders in the implementation of leisure activities as forms of engagement that promote social interaction in dementia and eldercare.

To ensure the relevance of the data to the research question, we re-examined all interview transcripts for discussions explicitly mentioning board games and non-digital leisure activities as forms of engagement that foster social interaction.

To ensure rigor and minimize bias, co-authors A and B independently conducted an inductive qualitative thematic analysis (Thomas, 2006) solely on this subset of data using Atlas.ti 8.3 for Macintosh (Atlas.ti Scientific Software Development GmbH). To ensure the analysis aligned with the research question of the present study, the dataset was thoroughly reviewed and recontextualized by author A.

Regular consensus meetings were held to resolve discrepancies and refine themes, and cross-checks with the original dataset and interview transcripts ensured the validity and trustworthiness of the interpretations. Subsequently, coded transcripts were grouped into higher-level categories and themes specifically addressing the implementation of non-digital leisure tools (e.g., board games) in dementia and elder care. Any ambiguity regarding differences in coding was settled during a consensus meeting with co-author D.

## 3. Results

### 3.1. Overview

Stakeholders provided insights into the decision-making processes and implementation of non-digital leisure activities to promote social interaction in dementia and eldercare settings. A thematic analysis of stakeholder interviews identified three key themes: (1) inclusivity, emphasizing the importance of adapting activities to meet diverse cognitive and physical needs; (2) usability, which highlights the practical considerations of implementation, including ease of use, privacy concerns, and engagement monitoring; and (3) context, which captures the external factors such as budget constraints, resource availability, and institutional priorities that influence the feasibility of these activities in dementia and eldercare settings (see Table 2).

#### 3.1.1. Inclusivity

Participants cited the positive aspect of non-digital tools in bringing joy and comfort by facilitating social interaction for older adults with or without dementia. Particularly for individuals with more severe cognitive impairment or physical comorbidity, therapeutic non-digital interventions can be more acceptable as they provide opportunities for engagement. Several stakeholders emphasized that these activities should not only be engaging on an individual level but also create a shared social experience that fosters communication and connection with others.

In terms of board games, one participant specifically highlighted that adopting them as a leisure activity might be easier for older adults as it is “familiar” and is “closer to the comfort zone” when addressing their (social) needs. Stakeholders emphasized the importance of a sense of belonging and identity. While non-digital-based leisure activities such as board games are often entertaining, their familiarity and accessibility can make them particularly meaningful for older adults. Participants indicated that not only do non-digital tools have a high potential to support well-being, but they are also necessary.

*“I think they are really important, especially when you are managing really affected profiles in Alzheimer’s or dementia. In the past, I mean, we decided to be 100% online. But if they are really affected, you definitely need offline materials. Some kind of printed solutions, so they print some of the tasks* etc.*”*

—industry professional (Spain)

Generally, stakeholders emphasized that non-digital leisure activities must be adaptable to the cognitive and physical abilities of older adults, ensuring that all residents, including those with advanced dementia, can participate. The need for familiar and culturally relevant activities was highlighted as essential for sustaining social engagement.

#### 3.1.2. Usability

Participants cited privacy and a “safe environment” as positive aspects of non-digital tools, particularly in group settings where social interaction naturally emerges. It was noted that structured activities, such as board games, provided a socially engaging environment for older adults to interact with peers, reducing feelings of loneliness and isolation. However, stakeholders discussed practical considerations that affect the successful implementation of these activities. Addressing potential problems, as well as tracking user engagement and satisfaction, requires continuous monitoring. Subsequently, challenges such as staff involvement, monitoring participation, and ensuring consistent engagement were noted. Some stakeholders suggested that the success of these activities in promoting social interaction depends on the extent to which they are incorporated into daily routines and encouraged by care staff. Combining digital reminders with non-digital activities was suggested as a way to enhance adherence. Participants mentioned that combining non-digital and digital tools might be a potential solution for providing more inclusive but also easier-to-monitor support, ensuring that individuals regularly participate in (socially interactive) leisure activities. In this context, sending notifications to those with dementia and their caregivers might be an efficient strategy to promote using non-digital tools:

*“In fact, one of the reminders that we want to implement is reminding people to do things that are part of the offline. So reminding them that their crossword puzzle is, you know, in the bookshelf or on the kitchen table so that they can do those little joys and comforts.”*

—industry professional (United Kingdom)

#### 3.1.3. Context

This theme captures practical insights from stakeholders on how non-digital leisure activities can be effectively integrated into dementia and eldercare settings. Apart from the COVID-19 pandemic that has massively influenced the distribution and implementation of non-digital tools in nursing homes, several participants noted that the challenges in distributing and implementing these tools would depend on the context and scope of the product. It was noted that leisure activities often receive less priority in care planning despite their potential to enhance social engagement and improve mental well-being. In particular, stakeholders emphasized that institutional support is necessary to ensure that non-digital leisure activities are recognized not only as recreational but also as valuable tools for enhancing social interaction and well-being among residents.

Stakeholders emphasized the importance of involving activity coordinators in nursing homes, balancing digital and non-digital options, and securing institutional support to ensure sustainability. Stakeholders emphasized the role of activity coordinators in organizing leisure activities and opportunities to promote active participation rather than passive entertainment. Specifically, for activity coordinators of nursing homes, it is important if the product supports leisure activity or care. While some stakeholders used the term ‘entertainment,’ the discussion focused on activities that promoted engagement and interaction, rather than passive recreational options:

*“I think one of the things to bear in mind in terms of the way that care homes are set up is that usually, they have people that are designated activity coordinators. So they will be responsible for, well, essentially the activities and entertainment of the residents in that care home, so as I said, we’ve got to differentiate leisure activity and the actual delivery of care, right?”*

—researcher (United Kingdom)

One participant suggested that the aim and the context of non-digital tools might play a role in their implementation in nursing homes:

*“We’ve got cohorts of patients in inpatient units that have activity coordinators and people to do these things, but not a lot of specific sort of well-being tools or something around cognition and cognitive stimulation. So I think anything that could be used in activities for patients, particularly in inpatient areas, but at home or wherever they may be, is really important. We don’t have a lot of that really”*

—policy officer (United Kingdom)

## 4. Discussion

This study provided additional insights through secondary analysis from semi-structured interviews conducted with stakeholders with expertise in dementia and eldercare. The focus was on investigating stakeholders’ perspectives regarding the implementation of non-digital leisure activities in dementia and eldercare settings. The results indicate that stakeholders recognize inclusivity as a key factor in implementing non-digital leisure activities, as activities must be adaptable to various cognitive and physical abilities. However, usability concerns, such as staff availability and engagement monitoring, were frequently raised, highlighting barriers to sustainability. Stakeholders also identified institutional constraints, including budget limitations and policy priorities, as critical challenges in integrating these activities into dementia and eldercare settings. These findings suggest that future implementation efforts should emphasize structured planning, staff training, and increased institutional support to enhance the impact of non-digital leisure activities.

In line with previous research, stakeholders in this study highlighted that the successful implementation of non-digital leisure activities relies on their meaningfulness to individuals (Russell et al., 2022). These activities should go beyond mere entertainment, promoting engagement, interaction, and personal fulfillment. To maximize their impact, leisure activities should be designed to align with older adults’ interests, abilities, and cultural backgrounds (McGuinn & Mosher-Ashley, 2001). While entertainment value was recognized, stakeholders highlighted that meaningfulness, such as promoting the sense of identity, cognitive stimulation, and social interaction, was a critical determinant of sustained engagement. These findings highlight the need for a person-centered approach, tailoring activities to individual needs to enhance their social (and therapeutic) value.

### 4.1. Promoting Inclusive Leisure Activities in Dementia and Eldercare

Psychological and hedonic leisure activities, such as board games and storytelling, have been associated with enhanced social well-being and a sense of belonging among older adults (Leversen et al., 2012; P. Chen et al., 2022). These activities provide opportunities for shared experiences and can encourage engagement in long-term care settings. While previous studies have highlighted their benefits, limitted research has explored the perspectives of key stakeholders on how these activities are implemented in dementia and eldercare settings.

The study findings underscore the significance of supporting innovations in both digital and non-digital leisure activities within dementia and eldercare. Blending these innovations, such as offering both digital and non-digital versions of activities, can provide an inclusive approach to engaging a broader audience and promoting social interaction, leisure, and meaningful activities (Gauthier et al., 2019). However, participants remained skeptical regarding implementation, particularly concerning the transition of non-digital leisure products into practice. They identified several barriers, notably the challenge of evaluating the effectiveness of self-reported outcomes and measuring user engagement or satisfaction with non-digital leisure resources, especially within the context of nursing homes. However, they also highlighted the importance of establishing clear boundaries between leisure and care as a facilitating factor. It is noteworthy that stakeholders’ perspectives on non-digital leisure activities may introduce biases, particularly ageist assumptions about older adults’ interest or ability to engage, leading to passive care models. To counter this, leisure activity development could adopt a participatory approach, incorporating both stakeholder insights and direct input from older adults (Gaber et al., 2024). This is in line with previous research indicating that, while stakeholder views on feasibility and resources are valuable, they should complement rather than dictate leisure programming to ensure that activities remain diverse, engaging, and personalized (Healy et al., 2022).

Participants in this study suggested that future innovations in this field should adopt a broader, non-dementia-specific approach. However, it was also highlighted during the interviews that “...*If you get it right for dementia, you’ll get it right for everyone*,” pointing to the complex nature of dementia and the feasibility of taking a more comprehensive approach that could benefit a wider population, including both those with and without dementia, especially in nursing home settings (Christie et al., 2024).

### 4.2. Clinical Implications

Given that nursing homes face a significant challenge in expanding the availability of diverse and ongoing activities for residents with varying interests and needs (Tak et al., 2014), future research can enhance the significance of such initiatives by employing theory-guided approaches. In previous research, a person-centered board game was co-developed with and for people with dementia to encourage social interaction (Niedderer et al., 2022). These approaches can target a spectrum of common risk factors for social isolation among older adults with and without dementia living in communities and nursing homes (Hu et al., 2022). This may involve integrating theory-driven positive psychology constructs, life storytelling, reminiscence, and mindfulness into the product design to ensure generalizability and effective scalability across cultures (Akhter-Khan & Au, 2020).

Additionally, in the context of implementing psychological and hedonic leisure activities, it should be noted that too little or too much involvement can cause emotional burden or dissatisfaction. Stakeholders in this study raised concerns about staff involvement in organizing leisure activities, noting that resource limitations could reduce the feasibility of structured programs. To mitigate this, institutional policies should emphasize training care staff to facilitate non-digital leisure activities effectively, ensuring that implementation does not become an additional burden on already overextended personnel. Future research could focus on identifying optimal levels of engagement to maximize benefits while minimizing negative effects (Li et al., 2021). This approach enables an evidence-based assessment of how these activities contribute to enhancing social interaction, improving quality of life, and promoting adaptation among nursing home residents (Gauthier et al., 2019).

From a policy perspective, funding allocations should prioritize the integration of non-digital leisure activities into dementia care plans. Stakeholder input can be useful in determining the logistical feasibility of these activities, but direct engagement with older adults is crucial to ensure that decisions reflect their lived experiences and preferences. Policymakers should also prioritize adherence to and promotion of guideline-based involvement to prevent biases (McArthur et al., 2023), such as assumptions about older adults’ abilities, from influencing leisure programming in ways that may restrict or exclude participation.

In the context of nursing homes, previous research showed that greater support from the environment for engaging in leisure activities is correlated with a higher degree of self-determined motivation, which is strongly linked to better adjustment to nursing home living (Altıntaş et al., 2017). Thus, nursing homes should view psychological and hedonic leisure activities as an opportunity to cultivate a socially supportive environment, promoting relationships between staff and residents, as well as among residents themselves, particularly during leisure activities. This facilitates the integration of individuals into the group and promotes a smooth process of adaptation (Altıntaş et al., 2017).

By shedding light on the implementation of these non-digital leisure activities, developers can improve their design and implementation, making them more efficient, innovative, and practical for adopters residing in both community and long-term care settings. Furthermore, the insights obtained from this exploration might also inform policy decisions regarding resource allocation for leisure activities aimed at enhancing the social well-being of older adults.

### 4.3. Limitations

The study’s limitations include a small sample size of stakeholders, potentially limiting the generalizability of findings. Additionally, although stakeholders’ insights were grounded in their professional expertise and extensive interactions with older adults and caregivers, it is essential to recognize that stakeholders’ views may not fully align with the desires and preferences of older adults. Future research should prioritize incorporating older persons’ voices to ensure that leisure activities are designed and implemented in a truly person-centered manner.

The diverse backgrounds of participants provide valuable insights into cultural, systemic, and contextual differences. However, it is important to acknowledge that differences in healthcare systems, policies, and cultural norms between countries could also act as a limitation, as these factors may introduce variability that complicates the generalizability of the findings. Subsequently, the participant profile can represent a limitation, as several stakeholders, particularly researchers and industry professionals, were experts in technology. This may have influenced their perspectives on implementing non-digital tools, potentially introducing a bias toward viewing them as complementary or secondary to digital solutions. However, the inclusion of clinicians and policymakers provided a more balanced perspective, focusing on practical and systemic considerations of non-digital activities within real-world care settings. Future research should aim to include a broader range of stakeholders, including direct input from older adults and non-technology-focused professionals, to provide a more comprehensive understanding of implementing non-digital tools in dementia and eldercare.

Additionally, while a secondary analysis of qualitative data offers valuable insights, re-analyzing such data in a different context may influence the rigor of the analysis or result in a lack of first-hand knowledge of the data (Ruggiano & Perry, 2017). However, this research serves as an initial exploration into understanding the factors influencing the implementation readiness of psychological and hedonic leisure activities in dementia and eldercare. Thus, the findings of this study should be interpreted in light of its potential bias and limitations, and future studies are needed to validate and expand upon these findings.

## 5. Conclusions

In conclusion, this study explores the implementation of psychological and hedonic leisure activities aimed at enhancing the social well-being of older adults. Social isolation poses a significant risk to the health of older adults, and in continuing the digitalization of the healthcare system and society at large, there is a risk of digital divide and exclusion for vulnerable populations who are in need of social support but lack experience with and knowledge of digital tools.

Our findings offer recommendations for the further development of non-digital leisure activities. Exploring possibilities to integrate these activities into digital resources could serve as a promising and inclusive alternative for addressing the leisure and well-being needs of older adults. Future research should take into account both the potential harms of the emerging digital divide and the potential benefits of innovations in non-digital tools.

## Figures and Tables

**Table 1:** Participant backgrounds and areas of expertise.

Interviewee Background	Area of Expertise	Country
Researcher	Technology, mental health, and dementia	Canada
	Technology design and dementia	United Kingdom
	Health apps and standardization	Spain
Industry Professional	Operating and managing a start-up on incorporating technology into dementia care	United Kingdom
	Sales and marketing management for a dementia technology company	France
	Owner of an eHealth intervention for dementia and business consultant at a multinational company	Netherlands
Clinician	Occupational therapist in dementia	Netherlands
Policy	Local digital healthcare implementation	Netherlands
	Management and strategic operations of a local healthcare provider network	United Kingdom

**Table 2:** Thematic categories of stakeholder perspectives on non-digital leisure activities.

Themes	Explanations	Categories (Example of Answers)
Inclusivity: Ensuring Social Participation Through Non-Digital Leisure Activities	This theme addressed the extent to which non-digital tools can meet the diverse needs of older adults, including those with advanced dementia, multiple comorbidities, or varying preferences. Inclusivity emphasizes tailoring activities to individual abilities and backgrounds, ensuring they are accessible and meaningful for all participants.	- People with advanced dementia - Older age - Need - Comorbidities - Preference
Usability: Practical Considerations in Supporting Social Interaction	The theme focused on practical aspects of non-digital tools, such as the ease of implementation, privacy considerations, and the ability to monitor engagement and satisfaction.	- High privacy - Authenticity (joyful) - Difficult to monitor (customer service) - Accessibility - Beneficial when digital and physical activities are combined
Context: Institutional and Policy-Related Factors Affecting Social Interaction	This theme reflected the external factors influencing the implementation of non-digital leisure activities, such as budget constraints, available resources, and institutional priorities.	- Lack of suitable options - Aim of the product (leisure, care) - Budget - Policy and different implementation paths

## Data Availability

The data supporting the findings of this study are available upon request. The data are not publicly accessible due to restrictions, such as containing information that could compromise the privacy of research participants.

## Supplementary Materials

The following supporting information can be downloaded at: https://www.mdpi.com/article/10.3390/bs15030347/s1.
