# Supplementary material for: Close to the Comfort Zone: Stakeholders’ Perspectives on Implementing Leisure Activities in Dementia and Eldercare
Source: Behav Sci (Basel). 2025 Mar 12;15(3):347. doi: 10.3390/bs15030347 (PMC11939336; doi:10.3390/bs15030347)
Supplement: Supplementary file 1 [file behavsci-15-00347-s001.zip › behavsci-3390550-supplementary.pdf]

Supplementary Materials:

Interview guide used in the original study

NASSS domains	Interview questions
Condition	1. Generalisability: Is there a need for a dementia-specific implementation readiness checklist? Why (not)?
Technology	2. From your experience, does the checklist include the aspects that are important in eHealth?
	3. Which aspects are missing, that you would like to include or change?
Value proposition	4. What do users (people with dementia, informal carers, health care professionals) want out of a digital tools?
	5. What do implementing organisations want out of a digital tool?
Adopters	6. How much of a say do you have in implementing new tools?
	7. Who else is involved in this decision?
Organisation	8. Why would/wouldn't an organisation adopt digital tools?
Wider system	9. Which political, policy, legal, professional or sociocultural factors influence the implementation of digital tools?
Embedding and adaptation over time	10. How (and by whom) should this checklist be distributed?
	11. Would it need to be updated over time?
Non-digital tools	12- What comes to your mind when you think of the implementation of non-digital tools, like board games, that are meant to be enjoyable rather than therapeutic?
	13- Could you elaborate on whether there are distinct barriers between non-digital and digital tools?
